# Ocular Drug, Gene and Cellular Delivery Systems and Advanced Therapy Medicinal Products

**DOI:** 10.4274/tjo.32458

**Published:** 2018-06-28

**Authors:** Türkan Eldem, Bora Eldem

**Affiliations:** 1 Hacettepe University Faculty of Pharmacy, Department of Pharmaceutical Biotechnology, Ankara, Turkey; 2 Hacettepe University Faculty of Medicine, Department of Ophthalmology, Ankara, Turkey; 3 Hacettepe University Institute of Health Sciences, Department of Stem Cell Sciences, Ankara, Turkey

**Keywords:** Ocular delivery systems, ocular gene and cellular delivery systems, colloidal drug and gene delivery systems, advanced therapy medicinal products, national and international legislation

## Abstract

Due to recent advances in science and technology, when the products used in therapy are examined, ophthalmology has a priority in terms of research and development, preclinical and clinical studies of innovative drugs, medical devices and drug-medical device combination products. Liposomes, micelles, nanoemulsions, nanoparticles with colloidal structures and intraocular implants as sustained-release drug delivery systems have been developed to overcome the barriers to ocular applications, increase absorption, decrease metabolism and elimination and increase the residence time in ocular tissues and compartments. Studies are also ongoing in the area of advanced therapies using gene or cell-based systems which are high-risk products due to their complex structures. In this review, ocular drug, gene and cellular delivery systems and related products and developments in advanced therapy medicinal products are presented in respect to the definition of drug (medicinal product) and current changes in legislation.

## Introduction and Objective

The bioavailability of drugs may be attenuated or inhibited by various factors, including the anatomical structure of the eye, the tear film, the varying permeability of the corneal layers to the drug substances and physiological ocular barriers such as the conjunctiva, blood-aqueous barrier, vitreous and blood-retina barrier. In order to cross these barriers in the anterior and posterior segments of the eye and achieve therapeutic drug concentrations in the targeted region, delivery systems with different structures and compositions that provide controlled or sustained release are being studied and used for treatment.^1,2,3^

In the design, manufacture and application of delivery systems, the properties of the product are determined by the active substance(s) being carried, as well as the system itself, the purpose of treatment, the procedures and devices used and their optimization strategies. Criteria determined in these contexts enable the classification of the product as a drug, medical device, or a drug-medical device combination product and elucidate the path to be followed in the approval process. The objective of this review is to present the developments in ocular drug, gene and cellular delivery systems and related products (including liposomes, nanoparticles, microparticles, implants and advanced therapy medicinal products) which have completed research and development (R&D), preclinical and clinical studies and are being used for the treatment of ocular diseases, within the framework of the definition of drug (medicinal product) and current changes in legislation.

## Drug Definition and Legislation

The R&D and manufacturing stages of drugs involve conventional production methods in pharmaceutical technology as well as biotechnological manufacturing processes and advanced technologies in the field of pharmaceutical biotechnology. Delivery systems that can be prepared on a nanometric scale, such as liposomes, are currently being produced via nanotechnology. These systems are known in the field of pharmaceutics as colloidal dosage forms and have been used therapeutically for years in accordance with drug licensing processes. In addition to these, nanodelivery systems with different structures and compositions and high-risk drugs, medical devices and combination products that fall into the scope of advanced therapies are being developed.

All previous studies have among their objectives to provide patients with safe and effective drugs and products and to find solutions for untreatable diseases or those with unmet needs. To achieve this goal, it is necessary to demonstrate the quality, efficacy and safety of the drugs or products and ensure quality assurance in their life cycle through a process that begins from pharmaceutical development. For this reason, a risk-based approach with quality risk management, pharmaceutical good manufacturing practices (GMP) and a pharmaceutical quality system must be implemented during registration.^4,5,6,7,8,9^

The European Commission has updated the definition of medicinal product (drug) in the European Union (EU) legislation, taking into account advances in science and technology, the development of innovative drugs and products and their associated risks.^10,11^ With the change made to this definition, the classification of biological products, medical devices and combination products has changed. The Regulation on Advanced Therapy Medicinal Products (ATMPs) issued by the EU in 2007 changed the directive that applies to human medicinal products.^12^ According to these changes, drug substances with low molecular weight, recombinant proteins having high molecular weight, monoclonal antibodies and the cell itself were classified as medicinal product (drug), biological medicinal product, or biological drug depending on how they are processed.

Products covered by the ATMP Regulation include “somatic cell therapy medicinal products”, “gene therapy medicinal products” and “tissue engineered products” that are categorized as drugs and “combined ATMPs,” which constitute drug-medical device combination products. Similar products that were approved prior to the publication date of this regulation have been granted time for compliance with the new legislation.^12^

In the United States (US), the Food and Drug Administration (FDA) requires New Drug Applications in the approval process for risky biological and biotechnological products.^13^ The FDA has issued a separate guideline for investigational new drugs and biological license applications for preclinical studies of biological products.^14^

During these amendments to the pharmaceutical legislation, the EU changed its directives pertaining to medical devices and *in-vitro* diagnostic products with two new regulations published in the official journal in May 2017 due to the issues observed with medical devices.^15,16^ Countries have been granted time to implement these changes, which will affect the international circulation of medical devices and associated products. This necessitates updating the national regulations on medical devices in Turkey, which have been harmonized with those in the EU, within the granted transition period.^17,18,19^ In the meantime, the US FDA has issued new regulations on drug-medical device combination products.^20,21^

In a period when international regulation on drugs, medical devices and related products are constantly being amended, regulatory harmonization has been conducted in our country and updates to the legislation are made by the Turkish Medicines and Medical Devices Agency (TMMDA) of the Turkish Ministry of Health. These include regulations involving drugs and medical devices.^22,23,24^Although drug regulations include different definitions of medicine or medicinal product, the Regulation on the Safety of Drugs defines a drug as *“*any substance or combination of substances presented as having properties for treating or preventing disease in human beings, or which may be used in human beings with a view to restoring, correcting, or modifying physiological functions by exerting a pharmacological, immunological or metabolic action*”*. Thus, our legislation contains a definition of drug or medicinal product that is consistent with current international regulations.^25^ This definition was later referred to in other regulations and a similar definition is included for “human medicinal product” in the Regulation on Manufacturing Plants of Medicinal Products for Human Use published in October 2017. This regulation encompasses the quality assurance system and the GMP included therein.^26^ In addition, the GMP guideline states that production requires “the establishment of an effective pharmaceutical quality assurance system and the term pharmaceutical quality system is used for consistency with international terminology”.^27^ With these updates, the national legislation of Turkey continues to effect change in accordance with internationally accepted criteria in order to harmonize with international regulations.

**Figure 1:**
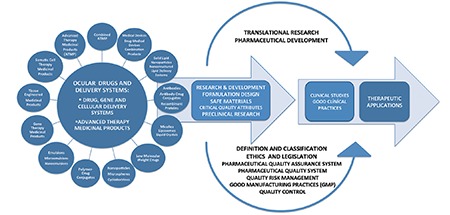
Basic requirements for the translation of ocular drug, gene and cellular delivery systems and advanced therapy medicinal products from the research and development and preclinal research stages to clinical investigations

As in all diseases, the approval process for ocular drugs and delivery systems is based on the structure, properties and intended use of the active substance and the delivery system containing it. In the course of developing safe and effective drugs and products and delivering them to patients, the first step is demonstrating the quality of the drug or product manufactured under GMP conditions.^4,5,6,7,8,9,10,11,12,13,14,15,16,17,18,19,20,21,22,23,24,25,26,27^ Considering the diversity of drugs (medicinal products), medical devices and combination products, this process requires legislation and definitions. Legal definitions are important because they enable the classification of a product and determine the route to be followed in R&D, preclinical and clinical trials. Starting from pharmaceutical development, the basic requirements for transition to clinical research and the critical parameters of the variety of drugs and products within the scope of ocular applications are shown in Figure 1. In these processes, it is important to know and validate the properties of the active substance, delivery system and the resulting drug or product in terms of design, composition, production and stability.

Like other drugs, all ocular drug, gene and cellular delivery systems, associated products and ATMPs that are produced under pharmaceutical GMP conditions within a pharmaceutical quality assurance system or pharmaceutical quality system, that are of proven quality and that have been tested for safety and efficacy in preclinical studies must be applied in clinical trials to evaluate their safety and efficacy in humans.^4,5,6,7,8,9,10,11,12,13,14,15,16,17,18,19,20,21,22,23,24,25,26,27^

## Liposomes

Liposomes are nanovesicular or microvesicular drug or gene delivery systems which range in size from 0.025 to 10 µm and contain a single lipid bilayer or multiple interwoven lipid bilayers. With structures consisting primarily of phospholipids and cholesterol, liposomes can carry hydrophobic drugs within their lipid layers and hydrophilic drugs in the interior aqueous compartment enclosed by the lipid bilayer. Conventional liposomes contain only phospholipids and cholesterol in their structure and polymer-coated liposomes are produced by adding PEGylated phospholipids (phospholipids chemically modified with polyethylene glycol) to this composition. Targeted liposomes can also be prepared by chemically modifying the surface of liposomes with targeting molecules. Cationic liposomes containing positively charged components are used as non-viral gene delivery systems. Cationic liposomes form complexes with and transport negatively charged antisense oligonucleotides, plasmids, nucleic acids, or small interfering ribonucleic acids. Liposomes are prepared using sterile production processes at laboratory or industrial scale.^3,28,29,30,31,32^ Numerous R&D and clinical trials have been conducted in which cationic liposomes were used as non-viral gene delivery systems but none of these products have completed clinical phase studies.^33,34^ Conventional, polymer-coated and targeted liposomes being used therapeutically were licensed through existing pharmaceutical legislation.^3^

The liposomal products commercially manufactured to date have included doxorubicin, daunorubicin, cytarabine, vincristine sulfate, irinotecan, amphotericin B, morphine sulfate, verteporfin, bupivacaine as active substances. In addition, hepatitis B and influenza vaccines having targeted liposome structures have been developed.^30^ The assessment reports and short product and labeling information of these liposomal drugs and vaccines are published by the legal authorities of the countries in which they are approved. Targeted liposomal vaccines that are part of liposomal systems have also been referred to in the literature as virosomes.^35,36^ In 2017, a product containing daunorubicin and cytarabine was approved by the US FDA as the first liposomal combination drug.^37^

Among the liposomal drugs, Visudyne^®^, a conventional liposome containing vertoporfin, is the first liposomal drug developed for the treatment of subfoveal choroidal neovascularization due to macular degeneration, pathological myopia, chronic central serous chorioretinopathy and choroidal hemangioma. Visudyne^®^ is administered by intravenous infusion, then the active substance is activated by laser application to the eye.^38^

Although liposomes developed for parenteral administration have been used therapeutically for many years, the number of liposomal drugs for ocular and intraocular administration have passed from R&D to clinical trial for ocular and intraocular applications is rather limited. Liposomal delivery systems containing different active substances have been examined in preclinical studies with experimental applications in the anterior and posterior segments of the eye and there are numerous studies and patents in the literature.^39,40,41^ Examples include conventional liposomes containing amphotericin B,^42,43^ gentamicin,^44^ clindamycin,^45^ 5-fluorouracil,^46,47^ cyclosporine A,^40,48,49^ tobramycin,^50^ norfloxacin,^51^ acyclovir,^52^ tacrolimus (FK506),^53^ indocyanine green,^54^ and timolol,^#*#ref55#*^# and polymer-coated liposomes^40^ containing cyclosporine A.

Liposomal drugs that have transitioned from preclinical research to clinical phase trials include latanoprost-loaded conventional liposomes developed for subconjunctival administration. A study on the subconjunctival administration of liposomal latanoprost to rabbits demonstrated reduction in intraocular pressure for 3 months.^56^ Phase 1 and 2 trials on the safety and efficacy of latanoprost-loaded liposomes in the treatment of ocular hypertension and primary open-angle glaucoma have been completed.^57,58^ The liposomal latanoprost developed through these studies has been patented.^59^

Liposomes are known to present challenges in terms of their structures, properties and stability compared to other colloidal delivery systems. In 2002, the US FDA issued a draft guideline on the manufacturing, controls, pharmacokinetic properties and bioavailability of liposomal drugs having complex structures. This guideline was updated and reissued as a draft in 2015.^60^ In addition, the EU European Medicines Agency (EMA) has published its views on data requirements for the production of liposomal drugs and on surface coating of nanodrugs.^61,62^ These documents explained that specifications vary depending on the formulation and manufacturing conditions of liposomal drugs and that critical quality attributes should include particle size, size distribution and morphology of the vesicular structure of a liposomal drug. They state that quality attributes will impact *in-vivo* pharmacokinetic and pharmacodynamic properties of liposomes, which will affect the efficacy and safety of the drug and the need for comparability studies was noted.^60,61,62^ The US FDA draft guideline and the EMA opinions contain important criteria that should be considered and met when liposomal drugs are manufactured by other companies after patent expiry. Therefore, comparability studies to demonstrate the quality, efficacy and safety of liposomal drugs and manufacturing liposomal drugs as nanosimilar drugs have gained priority. The aforementioned guideline and reflections also elucidate how to proceed for liposomal systems in the R&D stage.

**Figure 2:**
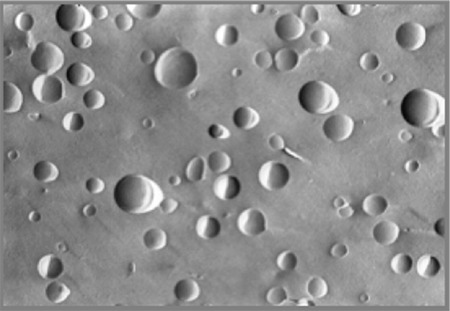
Morphological structure of polymer-coated liposomal cyclosporine A by freeze-fracture scanning electron microscope

Liposome particle size, vesicular structure and number of bilayers in the liposome membrane are among the analyses which are known to be critical and are evaluated in studies in the field of liposome technology. An example of this is a patent for liposomal cyclosporine A containing different phospholipids and phosphatidylethanolamine-PEG conjugates and prepared for ocular use with thin-film hydration followed by extrusion. According to this, polymer-coated liposomal formulations of cyclosporine A were developed and compared with conventional liposomal formulations.^40^ It was found that the aggregation observed shortly after preparation of conventional liposomal cyclosporine A did not occur with polymer-coated liposomal cyclosporine A formulations. The colloidal stability of liposomal cyclosporine A was provided by the steric coating formed on the liposome surface by the PEG component of the liposomes. An example of the unilamellar vesicular structure achieved with polymer-coated liposomal cyclosporine A is illustrated in Figure 2. It has been shown that polymer-coated liposomal cyclosporine A formulations have a z-average particle size (measured with laser light scattering) of 140-190 nanometers depending on the amount of drug present in the liposome composition and the structure, ratio and phase transition temperatures of the phospholipids and phosphatidylethanolamine-PEG conjugates and their polydispersity index varies between 0.08 and 0.20.

## Nanoparticles and Microparticles

Nanoparticles and microparticles are solid colloidal particulate systems that enable the controlled release of active substances which are adsorbed to the structure or dispersed or dissolved within the lipids or polymers forming the matrix. These delivery systems can be made with very different methods based on microencapsulation and polymerization technologies. Based on the size and structure of the resulting particle depending on the method used in the preparation or production and the solubility of components, they have been described as nanospheres, nanocapsules, microspheres, microcapsules, or micropellets.^63,64,65,66,67,68,69,70,71^ Matrix materials included in the composition of nanoparticles include albumin,^72^ chitosan,^73,34^ alginate,^75^ polylactic-glycolic acid,^76^ polyalkylcyanoacrylates,^77,78^ polymers such as hyaluronic acid coated poly-epsilon-caprolactone,^71^ lipids,^68,69,79,80,81,82^ and cyclodextrins.^83,84^ As a result of the studies carried out with nanoparticle and microparticle ocular delivery systems, there is no drug having particulate structure that is used in therapy.

Of the nanoparticulate drug delivery systems, Abraxane^®^ became the first to be approved by the FDA in 2005 after completion of clinical phase trials. This drug has colloidal dimensions, contains nanoparticle albumin-bound paclitaxel and is used parenterally for the treatment of metastatic breast cancer.^85^

Abraxane^®^ has been used in a phase 2 clinical trial for the treatment of inoperable intraocular melanoma.^86^ In addition, clinical phase trials have been started to evaluate the use of sulfur hexafluoride-lipid type A microspheres (Lumason^®^) for contrast in ultrasonography to diagnose cancer and evaluate brain perfusion.^87,88^ In another clinical trial in the EU, a phase 2 safety and efficacy study of ophthalmic dexamethasone nanoparticles in diabetic macular edema was launched in 2017.^89^

During the course of legislative changes, the US FDA issued another guideline in 2014 for applications classified as nanotechnology products within its jurisdiction. This guideline highlighted the need to consider how the properties of nanosized products (between 1-100 nanometers), their aggregates and surface-coated structures affect human health. Products in this guideline include drugs, biological products and medical devices.^90^

In addition, the EMA issued its position on the use of cyclodextrins as excipients. Although the document does not address cyclodextrin nanoparticles, it states that cyclodextrins enhance the ocular penetration of drugs and that 4% concentrations of a-cyclodextrin and 5% concentrations of randomly methylated b-cyclodextrin can be toxic in the corneal epithelium of rabbits. It also reported that a 10% solution of b-cyclodextrin sulfobutyl ether derivative and a 12.5% solution of b-cyclodextrin hydroxypropyl derivative had no toxic or irritant effect on rabbit eyes.^91^ Therefore, it is important to analyze the side effects and toxicity of cyclodextrin used in nanoparticles developed for ocular applications based on its structure, proportion and properties.

## Implants

In ocular implants, the active substance is contained in a reservoir and coated with polymeric membranes having different permeability. Release of the active substance from the implant at the desired rate and duration is designed according to the properties of the active substance and the polymers used. These systems were first developed as non-eroding implants; later, the use of biodegradable polymers enabled the design of eroding implants for treatment.^2,3^

The implants currently in use are delivery systems that contain low molecular weight drugs and can provide extended release of the active ingredient. The first of these, Vitrasert^®^, was developed as an intravitreal implant and contains ganciclovir.^92^ Later, Retisert^®^ and Iluvien^®^, which contain fluocinolone acetonide, were introduced.^93,94^ These implants are non-eroding and are surgically implanted and removed when necessary. In addition, the biodegradable implant Ozurdex^®^ is an intravitreal implant that provides sustained release of dexamethasone.^95^

Some systems reported in the literature are the subject of ongoing clinical studies. These include another delivery system containing live cells which enable the release of ciliary neurotrophic factor (CNTF) from genetically modified retinal pigment epithelium (RPE) cells.^96,97,98,99^ The release of protein drugs has been demonstrated from implants incorporating this system, which the manufacturer named “Encapsulated Cell Technology^®^” (ECT). The composition of ECT has live cells and an implant portion considered a medical device which allows the passage of proteins released from these cells into the biological fluids. Information about clinical trials being conducted with ECT is summarized in Table 1.^96,97,98,99,100,101,102,103,104,105,106^

In clinical trials evaluating ECT products in the treatment of retinitis pigmentosa, geographic atrophy and macular degeneration involving recurrent choroidal neovascularization, genetically modified RPE cells encapsulated in the NT-501 implant were applied to patients with different study protocols (Table 1)^96,97,98,99,100,101,102,103,104,105,106^

Results of studies conducted indicate that the rate of CNTF release can be controlled, the pharmacokinetic profile is appropriate, there is no passage into systemic circulation, the cells in the implant maintain their viability for the specified duration and there is no antibody formation against CNTF or the cells. However, it was reported that patients with geographic atrophy did not show statistically significant improvements in visual acuity and vision was only preserved in the group that received a high dose.^96,97,98,99^ The clinical research registry shows that another trial regarding the treatment of macular degeneration has been discontinued, while a phase 2 clinical trial initiated for the treatment of glaucoma continues.^105,106^ An article on the long-term (60-96 months) follow-up of retinitis pigmentosa patients who received ECT implants reported no signs of efficacy resulting from treatment.^107^

Because it encapsulates genetically modified cells in an implant and releases human neurotrophic factor into the eye via a semipermeable membrane, the EMA considered this ECT product a “cell-based drug delivery system”. The EMA legally classified the product based on EU regulations on medicines and ATMPs.^11,12,108^According to this, it was stated that the CNTF released from the genetically modified live cells has the properties of a drug active substance and the capsule with the semipermeable membrane that enables drug release and the polymeric scaffold on which the cells grow are medical devices integrated into the product. As a result of this evaluation, the product contained both drug and medical device components and was classified as an ATMP and “combined gene therapy medicinal product”. This classification was made based on the fact that the release of the active ingredient CNTF from the implanted system was enabled by genetically engineering RPE cells through biotechnological methods.^11,12,108^ Genetically altered RPE cells that release CNTF have received “orphan drug” status.^109^

## Advanced Therapy Medicinal Products

ATMPs include gene- and cell-based drugs and their combinations with medical devices.^12^ As stated in the legislations section, when human tissue and cell-containing gene and cell-based products for which the US FDA requires new drug applications and the ATMPs defined by the EU are considered in terms of their characteristics, it is observed that the same principles and criteria apply for ensuring their quality, efficacy and safety.

In order to enable the therapeutic use of safe and effective cell-containing drugs, the US introduced the “Regenerative Medicine Advanced Therapy” (RMAT) designation in the 21^st^ Century Cures Act enacted in 2016. The act describes these as a drug that is *“a regenerative medicine therapy, which is defined as a cell therapy, therapeutic tissue engineering product, human cell and tissue product, or any combination product using such therapies or products”*. According to the definition in the 21^st^ Century Cures Act, a drug is eligible for RMAT designation if it is *“intended to treat, modify, reverse, or cure a life-threatening disease or condition”*. The final requisite is that *“preliminary clinical evidence indicates that the drug has the potential to address unmet medical needs”*.^110^ The cellular drugs defined by this act do not include human cell-tissue products that have been used with minimal manipulation in routine treatment for many years through transplantation or transfusion.^110^ Drugs defined as RMAT or with RMAT designation according to this act are ATMPs and orphan drugs classified by the EU as ATMPs.

The four classes of biological medicinal products according to the EU ATMP Regulation have recently been listed on the EMA website as “somatic-cell therapy medicines”, “gene therapy medicines”, “tissue-engineered medicines” and “combined ATMPs” (http://www.ema.europa.eu). With developments in advanced therapies, efforts are ongoing to ensure international harmonization of the content and terminology used for drugs or medicinal products in the legislation of the EU and USA.

In the ATMP regulation, gene therapy medicinal products are described as biological medicinal products that *“contain recombinant nucleic acids or genes administered to humans for treatment, diagnosis and prevention”*. Somatic-cell therapy medicinal products are defined as *“products obtained from cells or tissues that are substantially manipulated to alter their biological characteristics, physiological functions, or structural properties and are not used for the same essential functions”*. *“Products that contain engineered cells or tissues and that are administered to humans for the purpose of repairing, regenerating, or replacing human tissue”* are designated as tissue-engineered medicinal products. *“ATMP that contain one or more medical devices as an integral part”* are called combined ATMP.^12^

In Turkey, ATMP are included in the “*Regulation on Registration of Medicinal Products for Human Use”*.^22^ In a guideline on the clinical research of ATMP, ATMP are defined as *“tissue- and cell-based human medicinal products classified as gene therapy medicinal products, somatic-cell therapy medicinal products, tissue-engineered medicinal products and combined advanced therapy medicinal products”*.^111^ In brief, ATMPs, which are defined as high-risk products in international regulations, are included within the scope of medicines in Turkish legislation in accordance with international principles. In addition, information on the manufacturing conditions of ATMP obtained from human tissues and cells is included in the *“Good Manufacturing Practices  (GMP) Guide for Manufacturing Plants of Human Medicinal Products”* updated by the Ministry of Health TMMDA, in accordance with the change in legislation.

In the EU, the multidisciplinary Committee for Advanced Therapies has been established within the EMA for ATMP and the committee considers applications, presents opinions and performs classification and certification procedures.^12^ The ATMP approved in the EU to date include a drug with the trade name Holoclar^®^ which was developed for ocular administration. Holoclar^®^ uses the patient’s own limbal stem cells, which are expanded and differentiated in culture to yield corneal epithelial cells. It has been reported that this biological medicine, which was conditionally licensed in the EU in 2015 within the framework of legislation, is the first stem cell-based ATMP. Holoclar^®^ is classified as a tissue-engineered medicinal product within the ATMP category.

It has been stated that although the active substance in the composition of Holoclar^®^ is human corneal epithelial cells, there are also stem cells in its structure. Holoclar^®^ is used in adults for corneal regeneration in cases of severe limbal stem cell deficiency and burns, including chemical burns and has orphan drug status. Administered by implantation, Holoclar^®^ is the equivalent of a transparent, circular live tissue containing 79,000-316,000 cells/cm^2^; the cells presented to treatment are found on a support layer of fibrin in transport medium.^112^

In addition, clinical research is being done with gene therapy medicinal products within the scope of ATMP. In one of these clinical trials, recombinant adeno-associated viral vector carrying human mitochondrial ND4 gene was classified as a gene therapy medicinal product (rAAV2/2-ND4). This orphan gene therapy medicine is administered intravitreally as a single dose to patients with Leber’s hereditary optic neuropathy and a clinical trial investigating of its efficacy is in progress.^113^

Clinical studies of gene therapy medicinal products initiated in the EU and USA include trials of retinal gene therapy providing AAV2 viral vector-mediated Rab escort protein-1 expression developed for the treatment of choroideremia. This research investigates the efficacy and safety of this gene therapy medicine, which is administered to patients subretinally as a single dose.^114,115^

In addition to this, the EMA in the EU classifies a large number of products within the scope of ATMP. These also include RPE cells obtained as a result of manipulating induced pluripotent stem cells. In the EU, RPE cells obtained through the differentiation of induced pluripotent stem cells have been classified as tissue-engineered products and medicines, based on their administration for regeneration, repair, or replacement in retinal degenerative diseases.^116^ RPE cells classified as drugs in the ATMP group are among the most studied and important cells in R&D in the development of retinal drug and gene delivery systems.^117^

In 2017, results were published of a clinical trial launched in 2013 in collaboration with Japan’s National Research Institute (RIKEN) for the use of RPE cells obtained from stimulated pluripotent stem cells for the treatment of age-related macular degeneration. The report stated that six patients were initially recruited for the study but a mutation was detected in the cell property analyses of the second patient and the trial was discontinued without performing the procedure in the second patient. In previous animal studies conducted with RPE cells obtained for this trial, the cells passed the tests related to tumorigenic properties but the procedure was not performed in the second patient due to the potential risks.^118^ Another document on the RIKEN website stated that after the trial launched in 2013, the Regenerative Drug Safety Act was enacted in Japan in 2014 and that the trial was discontinued due to insufficient time to complete the study (http://www.riken-ibri.jp/AMD/img/20151125en.pdf).

## Conclusion

From studies of drug delivery systems starting with liposomes, which are known to have an extended development and approval process, we have reached far more advanced stages today, where the cell itself is a drug, biological medicinal product, or advanced therapy product, or is given advanced therapy medicinal status in regenerative medicine and defined as a regenerative medicine. In this process, dosage forms such as liposomes and nanoparticles, known as colloidal delivery systems, are now referred to as “nanodrugs” or “nanopharmaceuticals” and fall within the field of nanotechnology. Studies are ongoing in the development of new nanodrugs for the treatment of eye diseases with different products and ocular implants are being used in therapy. Studies on ATMP and systems containing cells that enable the release of drugs with high molecular weight continue and the treatment of ocular diseases remains the priority.

This process in which various nanodrugs, gene and cellular delivery systems and ATMPs all involving their own risks are developed and in ongoing clinical research, involves a period of change and harmonization among national and international legislation. Compliance with national and international regulations is of utmost importance in the development of high-risk drugs obtained from engineered cells that are promising for the treatment of chronic diseases or untreatable eye diseases. Manufacturing these products within a pharmaceutical quality assurance system during development stages is a critical step toward faster transition from R&D to the clinic. This requires multidisciplinary research teams and the establishment of infrastructure with GMP conditions that meet the legal requirements of pharmaceutical quality systems.

## Figures and Tables

**Table 1:**
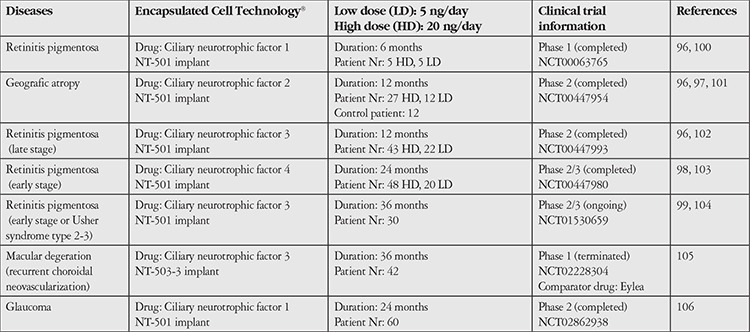
Information about the clinical studies related to the Encapsulated Cell Technology® products providing ciliary neurotrophic factor release from genetically modified retinal pigment epithelium cells
